# Automatic measurement of beam‐positioning accuracy at off‐isocenter positions

**DOI:** 10.1002/acm2.13844

**Published:** 2022-11-24

**Authors:** Tomohiro Ono, Takahisa Kido, Mitsuhiro Nakamura, Hiraku Iramina, Ryo Kakino, Takashi Mizowaki

**Affiliations:** ^1^ Department of Radiation Oncology and Image‐Applied Therapy Kyoto University Kyoto Japan; ^2^ Department of Information Technology and Medical Engineering Human Health Sciences Graduate School of Medicine Kyoto University Kyoto Japan; ^3^ Kansai BNCT Medical Center, Osaka Medical and Pharmaceutical University Takatsuki Japan

**Keywords:** beam‐positioning accuracy, developer mode, off isocenter, Winston–Lutz test

## Abstract

**Purpose:**

This study performed an automatic measurement of the off‐axis beam‐positioning accuracy at a single isocenter via the TrueBeam Developer mode and evaluated the beam‐positioning accuracy considering the effect of couch rotational errors.

**Methods:**

TrueBeam STx and the Winston–Lutz test‐dedicated phantom, with a 3 mm diameter steel ball, were used in this study. The phantom was placed on the treatment couch, and the Winston–Lutz test was performed at the isocenter for four gantry angles (0°, 90°, 180°, and 270°) using an electronic portal imaging device. The phantom offset positions were at distances of 0, 25, 50, 75, and 100 mm from the isocenter along the superior–inferior, anterior–posterior, and left–right directions. Seventeen patterns of multileaf collimator‐shaped square fields of 10 × 10 mm^2^ were created at the isocenter and off‐axis positions for each gantry angle. The beam‐positioning accuracy was evaluated with couch rotation along the yaw‐axis (0°, ± 0.5°, and ± 1.0°).

**Results:**

The mean beam‐positioning errors at the isocenter and off‐isocenter distances (from the isocenter to ±100 mm) were 0.46–0.60, 0.44–0.91, and 0.42–1.11 mm for the couch angles of 0°, ±0.5°, and ±1°, respectively. The beam‐positioning errors increased as the distance from the isocenter and couch rotation increased.

**Conclusion:**

These findings suggest that the beam‐positioning accuracy at the isocenter and off‐isocenter positions can be evaluated quickly and automatically using the TrueBeam Developer mode. The proposed procedure is expected to contribute to an efficient evaluation of the beam‐positioning accuracy at off‐isocenter positions.

## 1 INTRODUCTION

Recently, the single‐isocenter technique has been widely used for treating multiple brain metastases.^1^, ^2^ Several studies have reported that the single‐isocenter technique is more efficient than the multi‐isocenter technique in terms of the treatment time and monitor units (MU) delivered. However, there are some concerns, such as the dose and positioning accuracy for targets at off‐isocenter positions.^3^, ^4^ Wack et al. evaluated the impact of isocenter shifts on the delivery accuracy during the irradiation of small cerebral targets.^4^ They found that mechanical deviations, including gantry and collimator deviations and table rotations, might adversely affect the treatment of small stereotactic lesions. Therefore, these effects on the treatment of brain metastases, particularly with small volumes, are significant, and quality control (QC) for appropriate positioning and dose accuracy is required.

To ensure the safety of off‐axis treatment using the single‐isocenter technique, quality must be ensured from the patient‐ and machine‐specific perspectives. From a patient‐specific perspective, rotational errors in target coverage have been evaluated as an effect of dose distribution.^5^, ^6^ Roper et al. examined the dose distribution accuracy of single‐isocenter multitarget stereotactic radiosurgery (SRS) and demonstrated that rotational/translational inaccuracies and increased distance from the isocenter lowered target coverage.^7^ Tsuruta et al. evaluated patient setup errors as a result of intrafractional head motion in SRS,^8^ and the effects are primarily patient specific and important when considering treatment margins.^9^ From a machine‐specific perspective, the American Association of Physicists in Medicine (AAPM) task groups 142 and 198 reported quality assurance (QA) for medical accelerators.^10^, ^11^ For off‐axis treatment using the single‐isocenter technique, there are various uncertainties to ensure the safety of irradiation, such as rotational effect and beam‐positioning accuracy.^5^, ^12^ Particularly, the margin for stereotactic irradiation of the brain is only a few millimeters, making it difficult to give an adequate dose without evaluating off‐axis positional errors.^13^ In this study, beam‐positioning error is defined as the coincidence of the radiation and mechanical isocenter.

Focusing on beam‐positioning accuracy, the AAPM and Radiosurgery Society (RSS) recommend that the accuracy should be within 1.0 mm for SRS and stereotactic body radiation therapy (SBRT).^14^ The beam‐positioning accuracy is typically evaluated at the isocenter position; however, it is also necessary to confirm the beam‐positioning accuracy at off‐isocenter positions.^12^, ^15^ To evaluate the beam‐positioning accuracy at off‐isocenter positions, Gao et al. performed an off‐isocenter Winston–Lutz (WL) test for SRS and SBRT.^12^ They used a WL phantom, which had steel spheres of 8 mm in diameter embedded at the center of a 6 cm plastic cube. The phantom was placed at distances of 0, 3, and 5 cm away from the isocenter under eight beam conditions (different gantry, collimator, and couch angles), and the WL test was performed with an electronic portal imaging device (EPID). They concluded that the off‐isocenter distance for multiple targets should be less than 6 cm and recommended the off‐isocenter WL test to be performed for SRS and SBRT using the single‐isocenter technique. However, the suggested procedure is time consuming because a phantom must be set up for each measurement. Therefore, a more efficient, easy, and quick procedure is required.

In this study, we performed a quick measurement of the beam‐positioning accuracy at off‐axis positions using the TrueBeam Developer mode (Varian Medical Systems, Palo Alto, CA, USA). In addition, the effect of the couch rotational error on beam‐positioning accuracy was evaluated. The proposed procedure is expected to contribute to an efficient QC for beam‐positioning accuracy at off‐isocenter positions.

## 2 MATERIALS AND METHODS

### 2.1 Experimental setup

**FIGURE 1:**
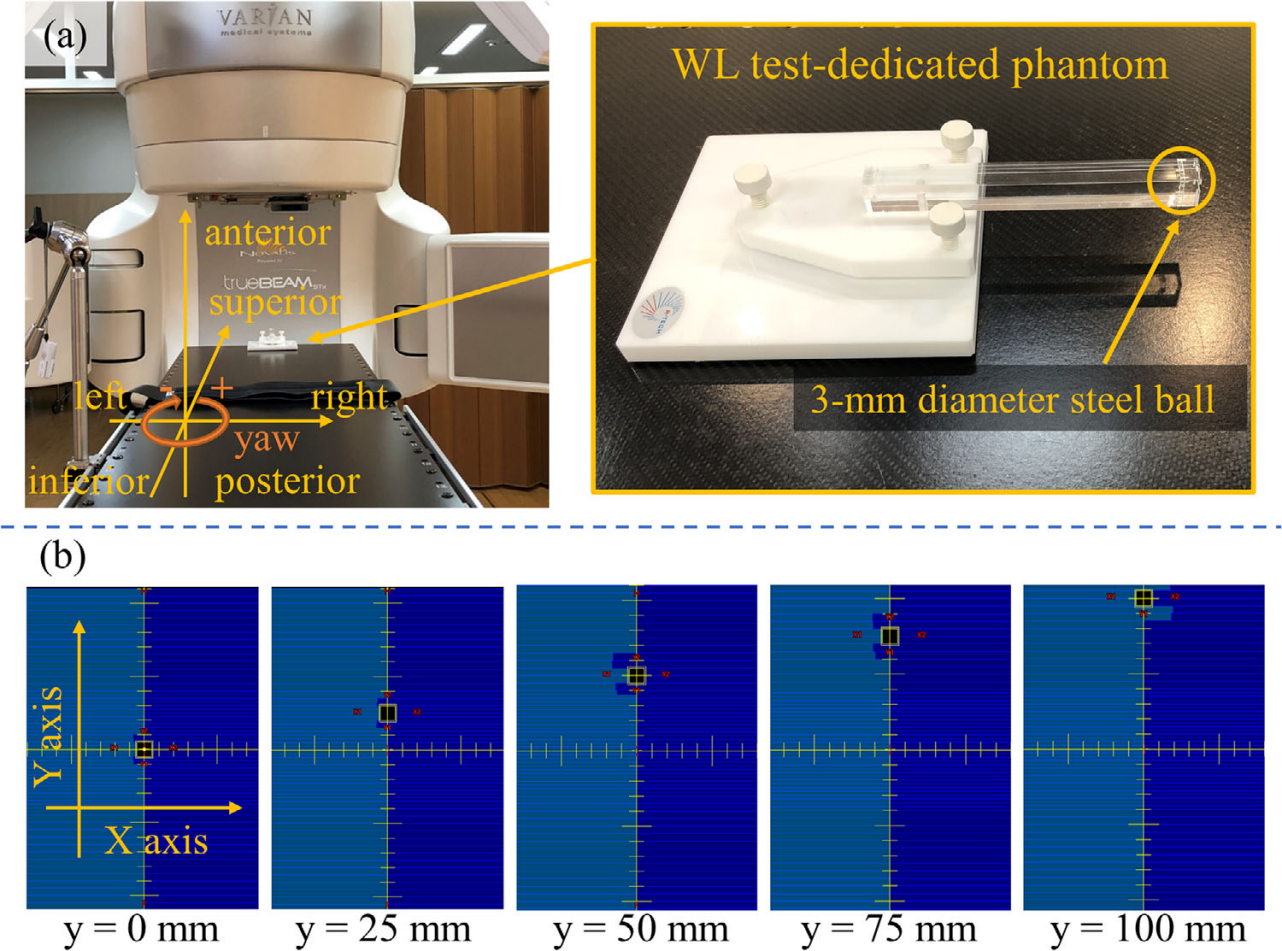
(a) Experimental setup for the evaluation of beam‐positioning accuracy; (b) WL test‐dedicated phantom placed on the couch and aligned at the isocenter using a room laser. The MLC‐shaped square fields were set at distances of 0, 25, 50, 75, and 100 mm away from the isocenter along each axis

Figure 1a shows the experimental setup for the evaluation of beam‐positioning accuracy. TrueBeam STx (Varian Medical Systems), with a 2.5 mm leaf‐width HD120 multileaf collimator (MLC) and carbon fiber couch (Brainlab, Munich, Germany), was used to evaluate the beam‐positioning accuracy. A WL test‐dedicated phantom with a 3 mm diameter steel ball (TM‐WINS: R‐Tech Inc., Tokyo, Japan) was placed on the couch top and aligned with the room laser beam. This study evaluated the beam‐positioning accuracy using the WL test and EPID‐based measurements.^16^ The EPID (aS‐1200, Varian Medical Systems) had a pixel pitch of 0.336 mm and image size of 1190 × 1190 pixels. Here, the EPID was set with a source‐to‐detector distance of 1500 mm and pixel size of 0.244 mm at the isocenter plane. MLC‐shaped square fields of 10 × 10 mm^2^ were created separately at the isocenter and off‐axis positions. Figure 1b shows an example of an MLC‐shaped square field. Here, MLC‐shaped square fields were created at each off‐axis position (distances of 0, 25, 50, 75, and 100 mm away from the isocenter position) in the Y direction in the beam's eye view (BEV). For each gantry angle, 17 patterns of MLC‐shaped fields were observed at positions of 0, 25, 50, 75, and 100 mm in the X and Y directions.

### 2.2 Automatic measurement using the developer mode

The TrueBeam Developer mode option was used.^17^, ^18^, ^19^ This mode allows for custom extensible markup language scripting and control of all components, including the couch, MLC, and beam delivery, which were programmed under the research conditions. It is possible to automatically perform a series of operations programmed by scripting, which helps improve the efficiency of the QC procedure. However, the TrueBeam Developer Mode is intended only for non‐clinical use and is not cleared for use on humans. In this study, gantry rotation and beam delivery were automated through the TrueBeam Developer mode. Small MLC‐shaped 10 × 10 mm^2^ fields automatically moved to each target position and 10 MU were delivered. The obtained images were analyzed using DoseLab software (Varian Medical Systems). In an obtained image, the iso‐valued curves were determined by the 50% and 90% thresholds, and the centers of these curves were identified. Here, the 50% and 90% thresholds were chosen based on the irradiated field size definition and the steel ball that was most easily detected, respectively. The beam‐positioning error was defined as the distance between the centers of the radiation field and steel ball. In this study, the measurement was performed assuming that the amount of couch movement was as indicated. The couch was moved such that the center of the steel ball was at the center of the MLC aperture. Thus, the resolution of the MLC was theoretically unaffected under the research conditions. The amount of couch movement was regularly evaluated to ensure that it was within 1.0 mm, which is the tolerance limit set by the AAPM‐TG 142.^10^

### 2.3 Evaluation of beam‐positioning accuracy at off‐isocenter positions

This study comprised two steps to perform the WL test at off‐isocenter positions using a single steel ball placed on the moving couch. First, the beam‐positioning accuracy at off‐isocenter positions was evaluated and compared with that at the isocenter position. The WL test was performed at the isocenter and off‐isocenter positions for four gantry angles (0°, 90°, 180°, and 270°) without couch rotation. Here, the WL test for the four gantry angles was defined as one cycle. The process of actual delivery of one cycle is presented in the Supporting Information S1. The off‐isocenter positions were 25, 50, 75, and 100 mm away from the isocenter along the superior–inferior (SI), anterior–posterior (AP), and left–right (LR) directions in the room's eye view (REV) condition (Figure 1a). This procedure was repeated three times, and 204 (17 positions × 4 gantry angles × 3 repetitions) images were obtained by the EPID with and without the TrueBeam Developer mode. Notably, the definitions of “X and Y directions in BEV” and “SI, AP, and LR directions in REV” were different. The positional relationship between the REV and BEV directions changes depending on the gantry angle. For example, when the gantry angle is 0°, the X and Y directions in the BEV correspond to the LR and SI directions in the REV, respectively; when the gantry angle is 90°, the X and Y directions in the BEV correspond to the SI and AP directions in the REV, respectively. Second, the effect of the rotational error of the couch on beam‐positioning accuracy was evaluated using the TrueBeam Developer mode. The WL test was also performed at the isocenter and off‐isocenter positions (25, 50, 75, and 100 mm away from the isocenter along the SI, AP, and LR directions) for four gantry angles (0°, 90°, 180°, and 270°) with couch rotations (0°, ±0.5°, and ±1.0°). The couch was rotated only along the yaw‐axis owing to the limited control of the carbon fiber couch (Brainlab) in the TrueBeam Developer mode. A total of 67 positions were measured using the TrueBeam Developer mode for each couch angle, and the images were obtained using the EPID. The measurements were performed three times on different days. The mean and standard deviation (SD) of the beam‐positioning error at each position were calculated.

## 3 RESULTS

### 3.1 Beam‐positioning error at the isocenter position

**FIGURE 2:**
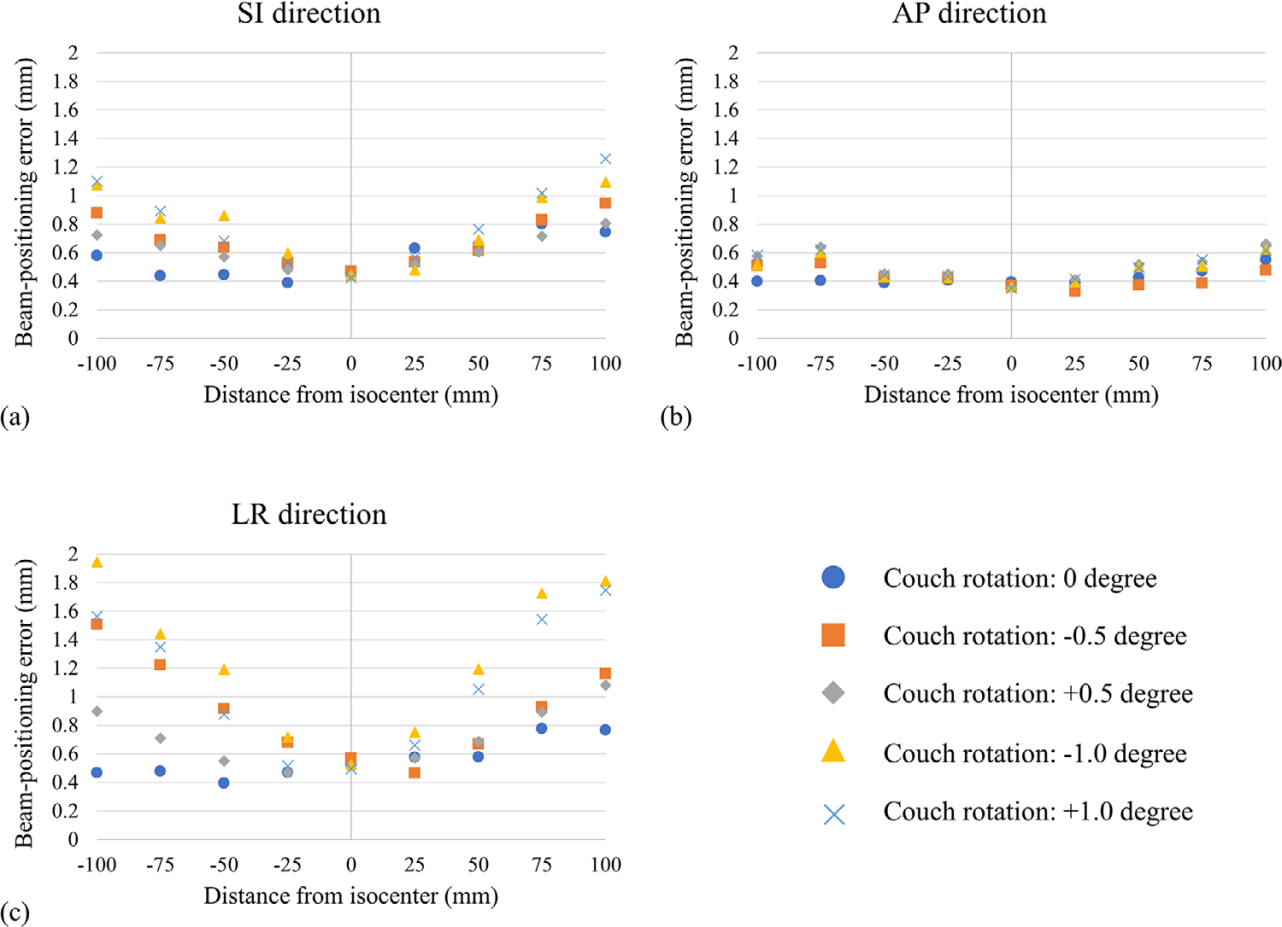
Relationship between the beam‐positioning error and distance from the isocenter for each couch angle. The graphs show the relationship in (a) superior–inferior (SI), (b) anterior–posterior (AP), and (c) left–right (LR) directions

The beam‐positioning error measurement took 2 and 20 min with and without the TrueBeam Developer mode, respectively, for one cycle. Figure 2 shows the beam‐positioning errors at the isocenter and off‐isocenter positions with a treatment couch rotation of 0°in the TrueBeam Developer mode for the SI, AP, and LR directions, as indicated by blue circles. The deviation of the beam‐positioning error was small in the AP direction. The mean ± SD of the beam‐positioning error was 0.46 ± 0.19 mm at the isocenter position. For the isocenter and off‐isocenter positions, the beam‐positioning errors were 0.49 ± 0.19, 0.50 ± 0.23, 0.58 ± 0.28, and 0.60 ± 0.28 mm (mean ± SD) at distances of 25, 50, 75, and 100 mm away from the isocenter, respectively. Therefore, the beam‐positioning error increased as the distance from the isocenter increased. In particular, we observed that the maximum mean positional error was 1.23 mm in the superior direction at a distance of 75 mm away from the isocenter.

### 3.2 Effect of couch rotation on the beam‐positioning error at off‐isocenter positions

Figure 2 shows the beam‐positioning errors with couch rotation at the isocenter and off‐isocenter positions in the SI, AP, and LR directions. The deviation of the beam‐positioning error was smaller in the AP direction with any degree of couch rotation than that in the SI and LR directions. For couch rotation angles of ± 0.5°and ± 1.0°, the beam‐positioning errors were >1 mm at distances of 75 and 50 mm away from the isocenter in the LR direction, respectively. Because a few radiation fields (at distances of 75 and 100 mm away from the isocenter for a couch rotation angle of ±1.0°) were outside of the EPID images, the beam‐positioning errors could not be evaluated. Table 1 lists the beam‐positioning errors under couch rotation angles of 0°, ±0.5°, and ±1.0°at distances of 0, 25, 50, 75, and 100 mm away from the isocenter. The mean beam‐positioning errors at the isocenter and off‐isocenter distances (from the isocenter to ±100 mm) were 0.46–0.60, 0.44–0.91, and 0.42–1.11 mm for couch angles of 0°, ±0.5 °, and ±1.0°, respectively. The largest mean beam‐positioning errors were observed at 100 mm under a couch rotation angle of 1.0°. We observed that beam‐positioning errors increased as the couch angle increased, especially in the SI and LR directions.

**TABLE 1:** Beam‐positioning error at each distance from the isocenter for each couch angle

	Distance from the isocenter (mm)									
	0		25		50		75		100	
Couch rotation angle (degree)	Mean ± SD	Max	Mean ± SD	Max	Mean ± SD	Max	Mean ± SD	Max	Mean ± SD	Max
−1.0	0.42 ± 0.24	0.80	0.53 ± 0.26	1.12	0.72 ± 0.39	1.47	0.99 ± 0.54	2.06	1.11 ± 0.66	2.38
−0.5	0.44 ± 0.23	0.77	0.49 ± 0.22	0.95	0.57 ± 0.26	1.15	0.69 ± 0.31	1.31	0.78 ± 0.38	1.59
0.0	0.46 ± 0.19	0.69	0.49 ± 0.19	0.94	0.50 ± 0.23	1.10	0.58 ± 0.28	1.23	0.60 ± 0.28	1.17
0.5	0.47 ± 0.27	0.93	0.50 ± 0.28	1.37	0.61 ± 0.36	1.62	0.76 ± 0.45	1.94	0.91 ± 0.53	2.22
1.0	0.44 ± 0.25	1.01	0.56 ± 0.33	1.51	0.80 ± 0.48	2.06	0.98 ± 0.59	2.50	1.05 ± 0.69	2.29

Beam‐positioning error is expressed in millimeters.

Abbreviations: Max, maximum; SD, standard deviation.

## 4 DISCUSSION

This study evaluated the beam‐positioning accuracy at off‐isocenter positions using the TrueBeam Developer mode with a measurement time of 2 min for each couch angle; we showed that the beam‐positioning accuracy can be measured quickly and automatically using this model. Furthermore, we confirmed that the beam‐positioning error increased as the couch rotation angle and distance from the isocenter increased. These findings are expected to contribute to the understanding of the beam‐positioning accuracy at off‐isocenter positions and development of efficient QC methods.

Recently, a new procedure for evaluating beam‐positioning accuracy at off‐isocenter positions has been developed. Capaldi et al. developed an integrated QA phantom for a single‐isocenter multitarget SRS.^20^ The phantom included four steel ball bearings with 3 mm diameters at off‐isocenter positions (maximum distance: 7 cm away from the isocenter) and evaluated the beam‐positioning errors between the planned and delivered positions for each steel ball. They found that the beam‐positioning error was within 1 mm at 7 cm away from the isocenter. StereoPHAN was developed by Sun Nuclear Corporation (Melbourne, FL, USA) as a multifunctional QA tool for SRS.^21^ StereoPHAN was designed such that several types of phantoms can be inserted, including the SRS MapCHECK (Sun Nuclear Corporation) or MultiMet‐WL Cube phantom (Sun Nuclear Corporation). These devices are efficient in verifying multiple off‐axis positional accuracies simultaneously for each gantry angle. In contrast, in the present study, we evaluated the beam‐positioning accuracy at off‐isocenter positions using a conventional WL test phantom via the TrueBeam Developer mode. In terms of measurement efficiency, the measurement time with manual estimation was 20 min and that with the TrueBeam Developer mode was 2 min. Thus, the automation of irradiation is expected to improve the efficiency of off‐axis positional accuracy evaluation. Furthermore, a combination of phantoms for off‐axis positional accuracy evaluation and irradiation automation will enable more efficient QA in the future.

In addition to the beam‐positioning accuracy, the effect of the rotational error in the single‐isocenter technique on SRS for multiple brain metastases has been reported by previous studies. Gao et al. stated that uncertainty due to the couch rotation angle is always present.^12^ Thus, couch rotation may result in the beam‐positioning error. Nakano et al. evaluated the rotational effect on dose distribution for spherical gross tumor volume (GTV) with diameters ranging from 1.0 to 3.0 cm.^5^ The GTV position vectors were rotated from 0° to 2.0°, and the distance between the target and isocenter ranged from 0 to 15 cm. They suggested excluding targets that are farther than 7.6 cm away from the isocenter when using a single‐isocenter technique to satisfy the tolerance value for all GTVs. Ezzell et al. evaluated the spatial positioning accuracy using the ExacTrac platform and cone‐beam computed tomography in the single‐isocenter SRS in patients with multiple brain metastases.^22^ A phantom with 12 steel balls was distributed up to 13.8 cm away from the isocenter. The positioning errors of each image guide were evaluated using seven gantry and couch angles. They found that both systems demonstrated a variation ranging from 0.9 to 1.1 mm in the 95% confidence limit at 7 cm from the isocenter. In the present study, we observed that the beam‐positioning error was within 0.92 mm with a couch angle of 0.5°at a range of 0 to 5.0 cm distance from the isocenter and confirmed that the accuracy was within 1.0 mm as recommended by the AAPM‐RSS.^14^ Based on various perspectives regarding the effect of rotational errors on dose distribution for the target and beam‐positioning accuracy, rotational errors should be detected and corrected using an image‐guided radiotherapy technique. Tsuruta et al. evaluated the correlation between intrafractional residual setup errors and the accumulation of delivered dose distributions in 72 consecutive patients with multiple brain metastases.^6^ They found no significant difference in the target coverage following the correction of the intrafractional residual error, despite the targets being far away from the isocenter. Thus, correction of the detected rotational errors ensures beam‐positioning accuracy.

The limitations of this study are presented as follows. First, beam‐positioning accuracy might have been affected by the EPID calibration. Generally, EPIDs are calibrated such that all pixels have the same response; however, in practice, the response of each pixel varies.^23^ When evaluating the beam‐positioning accuracy at off‐isocenter positions using EPID, the same MLC geometry is likely to show some variations in different institutions. Second, only the yaw‐axis for couch rotation was included. In general, the rotational error of the collimator and treatment couch affect the beam‐positioning accuracy. Gao et al. evaluated the beam‐positioning accuracy at off‐isocenter positions (within 9 cm away from the isocenter) with the gantry, collimator, and couch rotations and confirmed that the accuracy was within 1 mm.^15^ In the present study, the TrueBeam Developer mode with a carbon fiber couch (Brainlab) did not permit the control of couch rotation along the pitch and roll axes. To evaluate the beam‐positioning accuracy with the rotational effect, it is desirable to evaluate the effects of the couch and collimator rotations. Finally, although it is possible to verify the mixed positions off X and Y axes using the TrueBeam Developer Mode, beam‐positioning errors were evaluated only along the X and Y axes in the current study. Considering that a tumor does not necessarily lie on an axis, the situation of moving off the axis should be evaluated. Future studies should consider various scenarios, including mixed axes, large couch rotation for non‐coplanar beams, and collimator rotation, using the TrueBeam Developer mode.

## 5 CONCLUSION

We demonstrated that the beam‐positioning accuracies at the isocenter and off‐isocenter positions can be evaluated quickly and automatically using the TrueBeam Developer mode. Furthermore, beam‐positioning errors increased as the distance from the isocenter and the couch rotation increased. The proposed automatic procedure is expected to contribute to efficient QC for beam‐positioning accuracy at off‐isocenter positions.

## CONFLICT OF INTEREST

The authors declare no conflict of interest

## AUTHOR CONTRIBUTIONS

Tomohiro Ono and Takahisa Kido contributed equally to this work.

Tomohiro Ono, Takahisa Kido, Hiraku Iramina, and Ryo Kakino conceived of the presented idea and verified the analytical methods. Mitsuhiro Nakamura and Takashi Mizowaki helped supervise the project. All authors discussed the results and contributed to the final manuscript.

## Supporting information



Supporting Information.

## References

[1] Pan H , Cervino LI , Pawlicki T , et al. Frameless, real‐time, surface imaging‐guided radiosurgery: clinical outcomes for brain metastases. Neurosurgery. 2012;71(4):844‐851. 22989959 10.1227/NEU.0b013e3182647ad5

[2] Kamath R , Ryken TC , Meeks SL , Pennington EC , Ritchie J , Buatti JM . Initial clinical experience with frameless radiosurgery for patients with intracranial metastases. Int J Radiat Oncol Biol Phys. 2005;61(5):1467‐1472. 15817352 10.1016/j.ijrobp.2004.08.021

[3] Nakano H , Tanabe S , Utsunomiya S , et al. Effect of setup error in the single‐isocenter technique on stereotactic radiosurgery for multiple brain metastases. J Appl Clin Med Phys. 2020;21(12):155‐165. 10.1002/acm2.13081 PMC7769381 33119953

[4] Wack LJ , Exner F , Wegener S , Sauer OA . The impact of isocentric shifts on delivery accuracy during the irradiation of small cerebral targets—quantification and possible corrections. J Appl Clin Med Phys. 2020;21(5):56‐64. 10.1002/acm2.12854 PMC7286018 32196950

[5] Nakano H , Tanabe S , Yamada T , et al. Maximum distance in single‐isocenter technique of stereotactic radiosurgery with rotational error using margin‐based analysis. Radiol Phys Technol. 2021;14(1):57‐63. 33393057 10.1007/s12194-020-00602-2

[6] Tsuruta Y , Nakamura M , Nakata M , et al. Evaluation of correlation between intrafractional residual setup errors and accumulation of delivered dose distributions in single isocenter volumetric modulated arc therapy for multiple brain metastases. Phys Med. 2022;98:45‐52. 35490529 10.1016/j.ejmp.2022.04.012

[7] Roper J , Chanyavanich V , Betzel G , Switchenko J , Dhabaan A . Single‐isocenter multiple‐target stereotactic radiosurgery: risk of compromised coverage. Int J Radiat Oncol Biol Phys. 2015;93(3):540‐546. 26460996 10.1016/j.ijrobp.2015.07.2262 PMC4610743

[8] Tsuruta Y , Nakata M , Nakamura M , et al. Evaluation of intrafractional head motion for intracranial stereotactic radiosurgery with a thermoplastic frameless mask and ceiling‐floor‐mounted image guidance device. Phys Med. 2021;81:245‐252. 33485142 10.1016/j.ejmp.2020.12.019

[9] Rojas‐Lopez JA , Diaz Moreno RM , Venencia CD . Use of genetic algorithm for PTV optimization in single isocenter multiple metastases radiosurgery treatments with Brainlab elements. Phys Med. 2021;86:82‐90. 34062337 10.1016/j.ejmp.2021.05.031

[10] Klein EE , Hanley J , Bayouth J , et al. Task Group 142 report: quality assurance of medical accelerators. Med Phys. 2009;36(9):4197. 19810494 10.1118/1.3190392

[11] Hanley J , Dresser S , Simon W , et al. AAPM Task Group 198 report: an implementation guide for TG 142 quality assurance of medical accelerators. Med Phys. 2021. 10.1002/mp.14992 34036590

[12] Gao J , Liu X . Off‐isocenter Winston‐Lutz test for stereotactic radiosurgery/stereotactic body radiotherapy. Int J Med Phys Clin Eng Radiat Oncol. 2016;05(02):154‐161.

[13] Nataf F , Schlienger M , Liu Z , et al. Radiosurgery with or without a 2‐mm margin for 93 single brain metastases. Int J Radiat Oncol Biol Phys. 2008;70(3):766‐772. 18262089 10.1016/j.ijrobp.2007.11.002

[14] Halvorsen PH , Cirino E , Das IJ , et al. AAPM‐RSS Medical Physics Practice Guideline 9.a. for SRS‐SBRT. J Appl Clin Med Phys. 2017;18(5):10‐21. 10.1002/acm2.12146 PMC5874865 28786239

[15] Gao J , Liu X . Winston‐Lutz‐Gao test on the True Beam STx linear accelerator. Int J Med Phys Clin Eng Radiat Oncol. 2019;08(01):9‐20.

[16] Lutz W , Winston KR , Maleki N . A system for stereotactic radiosurgery with a linear accelerator. IntJ Radiat Oncol Biol Phys. 1988;14(2):373‐381. 3276655 10.1016/0360-3016(88)90446-4

[17] Iramina H , Nakamura M , Mizowaki T . Actual delivered dose calculation on intra‐irradiation cone‐beam computed tomography images: a phantom study. Phys Med Biol. 2021;66(1):015007. 33238248 10.1088/1361-6560/abcdeb

[18] Hirashima H , Nakamura M , Miyabe Y , et al. Quality assurance of non‐coplanar, volumetric‐modulated arc therapy employing a C‐arm linear accelerator, featuring continuous patient couch rotation. Radiat Oncol. 2019;14(1):62. 30971273 10.1186/s13014-019-1264-6 PMC6458733

[19] Fahimian B , Yu V , Horst K , Xing L , Hristov D . Trajectory modulated prone breast irradiation: a LINAC‐based technique combining intensity modulated delivery and motion of the couch. Radiother Oncol. 2013;109(3):475‐481. 24231240 10.1016/j.radonc.2013.10.031

[20] Capaldi DPI , Skinner LB , Dubrowski P , Yu AS . An integrated quality assurance phantom for frameless single‐isocenter multitarget stereotactic radiosurgery. Phys Med Biol. 2020;65(11):115006. 32235050 10.1088/1361-6560/ab8534

[21] Rose MS , Tirpak L , Van Casteren K , et al. Multi‐institution validation of a new high spatial resolution diode array for SRS and SBRT plan pretreatment quality assurance. Med Phys. 2020;47(7):3153‐3164. 32215929 10.1002/mp.14153

[22] Ezzell GA . The spatial accuracy of two frameless, linear accelerator‐based systems for single‐isocenter, multitarget cranial radiosurgery. J Appl Clin Med Phys. 2017;18(2):37‐43. 10.1002/acm2.12044 PMC5689957 28300379

[23] Parent L , Fielding AL , Dance DR , Seco J , Evans PM . Amorphous silicon EPID calibration for dosimetric applications: comparison of a method based on Monte Carlo prediction of response with existing techniques. Phys Med Biol. 2007;52(12):3351‐3368. 17664548 10.1088/0031-9155/52/12/003

